# Barriers to accessing appendectomy in the public sector health system in the Western Cape Province, South Africa

**DOI:** 10.1016/j.afjem.2024.10.221

**Published:** 2024-11-09

**Authors:** Johnelize Louw, Kathryn M. Chu, Peter S. Nyasulu, Réne English

**Affiliations:** a Centre for Global Surgery, Department of Surgical Sciences, Faculty of Medicine and Health Sciences, Stellenbosch University, Cape Town, South Africa; b Division of Health Systems and Public Health, Department of Global Health, Faculty of Medicine and Health Sciences, Stellenbosch University, Cape Town, South Africa; c Division of Epidemiology and Biostatistics, Faculty of Medicine and Health Sciences, Stellenbosch University, Cape Town, South Africa; d Division of Epidemiology and Biostatistics, School of Public Health, Faculty of Health Sciences, University of the Witwatersrand, Johannesburg, South Africa; e Department of Surgery, University of Botswana, Gaborone, Botswana

**Keywords:** Appendectomy, Appendicitis, Delays, Health, Care, Access

## Abstract

**Background:**

Appendectomy is the surgical treatment for acute appendicitis and barriers to timely care can lead to morbidity and mortality. In South Africa, patients experience delays during the stages of seeking, reaching, and receiving care. This study explored the perceptions and experiences of barriers to accessing appendectomy among patients, caregivers, and surgeons employed at selected public hospitals in the Western Cape, South Africa.

**Methods:**

A qualitative study was conducted through semi-structured in-depth interviews. Study sites comprised four public hospitals. The interviews were audio recorded, transcribed, and translated verbatim. Excerpts in the qualitative data were systematically categorised and themes were generated using inductive thematic analysis according to Braun and Clarke's methodology.

**Findings:**

The following themes were generated from the analysis 1) barriers related to late presentation to a healthcare facility and 2) barriers related to healthcare facility delays. Identified barriers were perceptions of appendicitis-like symptoms, the influence of beliefs, customs and culture on healthcare-seeking behaviour, personal and social positions and values, lack of knowledge of the health system, transport accessibility and affordability, delayed ambulance response time, and proximity of healthcare facilities. Key barriers experienced after presentation to a healthcare facility related to inter-facility transfers, surgical capacity, and the diagnostic and management capabilities of facilities.

**Conclusion:**

Participants in the study experienced, and perceived similar delays to accessing appendectomy to that reported in other African countries. Improved health literacy in communities could lead to timely healthcare-seeking behaviour for appendicitis and other emergency conditions. Efforts are needed to ensure access to affordable and available transport options, and healthcare facilities need to be better equipped to diagnose and treat appendicitis. This can be achieved through upskilling and augmenting human and other resources, which will require the support of the government and other relevant stakeholders.

## African relevance

• This study highlights the experiences and perceptions of delays in accessing appendectomy among patients, caregivers, and surgeons employed in the public sector in the Western Cape, South Africa.
• Participants provided a better understanding of public sector challenges encountered when seeking, reaching, and receiving appendectomy care which were similar to that reported in other African countries.
• Barriers reported were a lack of health literacy, socioeconomic factors, transport inaccessibility and availability, and a lack of resources within the health system to enable the timely diagnosis and treatment of appendicitis.
• Recommendations to improve access to appendectomy include improved health literacy in communities, and the need for strategies to upscale surgical care at lower-level facilities in the public health system such as surgical outreach.
• The lessons learned from this study regarding improved access to appendectomy might suitably be applied to similar environments in South Africa and other African countries.

## Introduction

Appendectomy is the surgical treatment for acute appendicitis [1]. Failure to diagnose and treat this condition timeously can lead to significant morbidity and mortality. Globally, South Africa has the highest incidence of appendectomy [2], with approximately 52 cases per 100,000 [[2], [3], [4], [5], [6]]. Understanding the efficiency of the health system requires metrics that can be used to measure the quality of the healthcare provided [7]. To this end, access to timely appendectomy can serve as a proxy measure for the performance and strength of surgical services within a country [[7], [8], [9], [10]].

In South Africa, appendectomy patients present late to healthcare facilities, and patients from rural areas tend to experience more severe disease than urban populations [[8], [9], [10], [11], [12], [13], [14], [15], [16]]. A study conducted at a rural referral centre in the Western Cape, South Africa, reported a median time from symptom onset until surgery of 60 (IQR: 41–85) hours, and from reaching the first facility to surgery of 25 (IQR: 13–42) hours [17]. This study also reported a 62 % post-operative complication rate, with two deaths [17]. In Kwa-Zulu Natal, South Africa, similar findings were reported [[8], [9], [10], [11], [12], [13], [14], [15], [16]]. Appendectomy patients from rural areas experienced double the median pre-hospital delay compared to those presenting from urban areas [10]. The overall complication rate in the rural vs urban group was 35 % with a mortality of *n* = 7 (3.5 %), and 11 % with a mortality of *n* = 1 (0.3 %), respectively [10].

Other studies suggest that the delays to appendectomy occur before and during healthcare facility presentation. In KwaZulu-Natal, patients needing appendectomy reported barriers to healthcare such as first visiting traditional healers, socioeconomic factors, and poor access to the health system [9,10,12]. After reaching a healthcare facility barriers to appendectomy can include limited hospital staffing, the lack of access to equipment and laboratory services, and inter-facility transfer delays [7,10,18].

Reasons for delays in the Western Cape, South Africa have not been well documented and require further study to inform health system policy and practice responses. This study explored the perceptions and experiences of patients, caregivers, and surgeons employed at selected public sector Western Cape hospitals, regarding the barriers to accessing appendectomy.

## Methodology

### Study design

This was an exploratory qualitative study.

### Study setting and participants

In South Africa, the health system is divided into a public and a private sector. Of the population, 15 % have medical insurance, which allows minimal waiting times for private-sector healthcare [19]. The remaining 75 % can pay out-of-pocket for private healthcare, or access subsidised care from the public sector [19]. In the public sector, hospitals are categorised as district, regional, and tertiary-level facilities.

### Sampling strategy

Participants were identified using purposive sampling. Appendectomy is the standard treatment for appendicitis, a disease that affects individuals of all ages and sexes. In the Western Cape, there are 33 district, 3 regional, and 2 tertiary-level hospitals. According to the District Hospital Service Package appendectomy performed by a general surgeon should be done at the district hospital. However due to a lack of surgical capacity at most district hospitals, this is not possible, and the majority of appendectomies are done at the regional and tertiary level.

Operative data are usually stored in logbooks. At least five facilities in the Western Cape have implemented electronic operative databases. This is helpful as operative logbooks are disposed of or moved to storage after five years. In addition, these logbooks can be damaged due to infrastructural challenges or natural disasters. Considering all of this, we purposively selected a district hospital that performs appendectomy (Eerste River (urban)), two regional hospitals (Worcester (rural) and New Somerset (urban)), and one tertiary hospital (Groote Schuur (urban)). According to the literature, to understand common perceptions and experiences among groups of homogenous individuals, 6 - 12 qualitative in-depth interviews are usually recommended and should be enough to reach data saturation [20,21]. We recruited the head of general surgery at each facility, and two additional surgeons to confirm that data saturation had been reached.

These study hospitals serve patients from low socioeconomic settings and three of the facilities have electronic operative databases which enable easier identification of appendectomy patients. As appendectomy is one of the most common procedures performed worldwide, we recruited three patients per facility, to ensure a sample size of at least 12 patients. After 12 in-depth patient interviews were done, data saturation were reached. We also purposively selected patients who had appendectomy in 2019, before the COVID-19 pandemic to avoid any pandemic-specific barriers from being reported. Furthermore, ensuring that time had passed between the interview and their procedure meant the patients had time to reflect on their care pathway and on their experiences of accessing appendectomy. Patients could only be included in the study if acute appendicitis was their primary diagnosis, and if they had the procedure in 2019.

### Recruitment process

The researcher (JL) emailed the head of general surgery if they indicated an interest in the study to formally invite them to participate and to determine a suitable date and time for the interview. If they were unavailable, they were asked to nominate another surgeon.

The healthcare providers assisted the researcher with patient recruitment. Due to ethical considerations and the Protection of Personal Information (POPI) Act, the researcher was not allowed to access the list of appendectomy patients. The healthcare providers at the study hospitals were asked to contact three appendectomy patients at a time, starting in January 2019. If any of the patients agreed to participate in the study, they were asked to provide times that would be convenient for the researcher to contact them. Therefore, if three attempts at contacting the participant on separate occasions, went unanswered, the healthcare provider would contact the next three appendectomy patients on the operative list for 2019. Recruiting participants from the same year for all the study hospitals minimised biases from provincial-wide events happening at different times in the year (such as COVID-19, nursing strikes, and medication stockouts).

No prior relationship was established between the researchers and the surgeons or patients.

### Data collection

Data collection was completed using semi-structured in-depth interviews. The interview guide was designed based on the Three Delays framework [22] and was piloted on a patient and surgeon, and revised accordingly. Interviews (11 patients, 1 caregiver, and 6 surgeons) were conducted throughout the year 2022 until data saturation were reached. All the interviews lasted between 30 – 180 min. Surgeons were interviewed in a room at their facility. Importantly all the surgeons who were approached agreed to participate in the study. Patient interviews were conducted by the primary researcher (JL) at a venue of their choosing. To ensure at least three patients were interviewed from each hospital, the healthcare providers contacted 21 patients overall. Two (9.5 %) of these patients declined to participate due to busy schedules, one (4.8 %) patient agreed but never showed up for the interview, and six (28.6 %) patients never answered the researcher's phone calls. Field notes were taken throughout the interviews. Participants were reimbursed for their time, inconvenience, and travel expenses if applicable. No repeat interviews were conducted and participants were not asked to comment on the transcripts due to their distribution and the difficulty with relocating the patients.

### Data analysis and management

Audio recordings of interviews were transcribed in sequence and were translated into English if necessary. Inductive reflexive thematic analysis (TA) according to Braun and Clarke's methodology [23] was used, as the analysis of the findings were reflective of the participants’ experiences and their perceptions of delays to accessing appendectomy [23,24]. The primary researcher (JL) coded the interviews, and the themes were finalised with input from the remaining co-authors (RE, KC, PN). Themes were identified at the latent level and the six phases of reflexive TA were followed: 1) data familiarisation, 2) initial code generation, 3) generation of initial themes, 4) theme review, 5) theme defining and naming, and 6) report production [24,25]. NVivo 12 software was used to manage the data.

### Researchers and reflexivity

This study was written up in line with the Consolidated Criteria for Reporting Qualitative Research (COREQ) [26]. The primary researcher is a young female, who has experience in qualitative research. She was not previously hospitalised. Participant interviews were conducted by her, with the help of a translator as needed. The co-authors are either experts in the surgical field or qualitative methodology and supported her accordingly. All the authors reside in South Africa.

### Ethical considerations

This study was approved by the Health Research Ethics Committee of Stellenbosch University (S21/02/031 (PhD)), the Health Research Ethics Committee of the University of Cape Town (460/2021), and the study hospitals (WC_202,104_030).

## Findings

### Demographics

In this section, the findings of 18 interviews (three patients and 1 – 2 surgeons per hospital) are reported. Overall, three patients, and two surgeons, were from rural communities. The surgeon and patients ages ranged between 34 – 57 years, and 21 – 71 years, respectively. Additional demographical information on the participants can be found in Supplementary Table 1 (Appendix 1). The barriers that were identified were the same across the surgeon and patient key informants. These findings were categorised into 1) barriers related to late healthcare facility presentation, and 2) barriers related to healthcare facility delays.

### Barriers related to late healthcare facility presentation

#### Patient perceptions of appendicitis-like symptoms

A dominant theme that was reported by the patients and the surgeons was that non-specific early appendicitis symptoms could be dismissed as temporary, self-limiting ailments for which over-the-counter medication was utilised. Symptoms were at times attributed to other underlying chronic conditions and treated as such.

“*My mother has arthritis and when the appendix happened, we thought it was a hip pain that shot up to her stomach. Because of her arthritis, she's on certain pain medication. The pain went away after I administered the paracetamol.”* (Caregiver 1)

*“The pain started three weeks to a month before the operation. I started getting stomach cramps and feeling uncomfortable, but it came and went… For three weeks, I was living on very strong analgesics. I think I was going through 30 tablets in four days. It reached the point where I could not bear the pain anymore and the medication stopped working.”* (Patient 3)

#### The role of tradition, culture, and beliefs on healthcare-seeking behaviour

In this study, initially purchasing traditional medicine was customary and the first choice for patients across racial groups. These choices were also linked to cultural beliefs regarding gender roles and healthcare-seeking behaviours as reported by some of the participants.

*“My mother-in-law was worried about me and gave me some herbs for stomach aches. After drinking it, nothing happened. The pain continued.”* (Patient 1)

“*I had no choice. Because we are Xhosa people, a man and a father are expected to be tolerant of pain or enduring, regardless of the circumstances.”* (Patient 2)

The fear of a suspected medical diagnosis and a poor prognosis based on one's family history of disease can affect when care is sought. Personal beliefs regarding the state of health and illness and its management also influenced how patients responded to their symptoms.

*“My father had colon cancer. The fear that I also had cancer was the key to me not seeking care immediately.”* (Patient 7)

The example provided below illustrates how the onset of abdominal symptoms was tolerated by the patient who could not believe that he was ill or needed medication, leading to a delay in presentation.

“*I am not a person that just goes to the doctor. I do not believe in drinking tablets; I believe that the body should heal itself.”* (Patient 1)

A dominant theme among the patients and the surgeons was that the perceptions and experiences of how patients, their friends, or family members were previously treated at public healthcare facilities influenced future healthcare-seeking behaviour. Furthermore, lower socioeconomic status community members perceived that hospital care was a terminal state and thus delayed seeking care until their symptoms were severe. As a result, many patients die after reaching a healthcare facility.

*“I do not want to go to this hospital because they do not listen to what you say to them. They only do their own thing without any explanation.”*

(Patient 4)

“*I think patients may be reluctant to access care if a family member died in the facility. And not even with their related illness, but just because their experience with the health system has not been a positive one.”* (Surgeon 2)

#### Patients’ personal and social positions and values

A patient's decision to seek care can be affected by their socioeconomic, and employment status. Surgeons reported a preference amongst patients to prioritise their jobs over their healthcare needs. In cases of extreme poverty, children may go to the extent of pushing their needs aside to accommodate the schedule of their parents.

*“I admitted a child yesterday with suspected appendicitis, who on Saturday morning started having pain. His caregiver is his grandmother, who works full-time on a weekend. He did not want her to leave work to attend to him or miss out on work. He only told her on Monday when she was available to take him to seek care.”* (Surgeon 1)

A dominant theme reported by the patients and the surgeons was a preference for seeking care within the private sector irrespective of patients’ financial abilities. This was based on the view that more individualised and faster care is provided within the private sector. However, as these same patients cannot afford to keep paying out-of-pocket for healthcare, they revert to the public sector eventually.

“*My mother wanted to go to her family doctor who was only available at 15:00. She will not go to another doctor and would rather wait in pain until* then*.”* (Caregiver 1)

*“When I arrived at the day hospital at 04:00, there was already a long line of people waiting. I got inside at around 09:00 or 10:00. At the day hospital, you will wait. They will tell you “No you are fine there's still a long line” even if you are in severe pain.”* (Patient 8)

#### Lack of knowledge of the health system

Patients lack or have limited knowledge regarding the health services structure and the corresponding provision within their local areas. In South Africa, it is common practice that patients require a referral letter to access further care in the public sector, particularly in non-emergency conditions. One of the surgeons and several patients shared this concern.

“*For R500, the general practitioner only tells patients to go to the hospital and if you do not have money, you cannot access this care.”* (Surgeon 3)

#### Transport accessibility and affordability

Financial barriers and transport inaccessibility can affect patients' ability to reach healthcare. A dominant theme among the patients and the surgeons was that patients would reach a healthcare facility sooner with alternative modes of transport than waiting for the emergency medical services (EMS) to escort them. However, financial reasons may delay when they can afford to access alternative transportation.

“*Until the symptoms are severe, many patients are going to weigh up whether they should spend their money on accessing healthcare or on food.”* (Surgeon 4)

Public taxis, buses, or trains may have multiple stops along the route before dropping the individual within walking distance from their desired location. Private transport is limited in its availability and patients are often overcharged. One of the patients noted that in her community, individuals are transported by a neighbour on request and can settle the outstanding amount when they receive their salary or wages. However, this inevitably leads to financial strain.

*“That pain was not nice. I was sitting in the taxi and you sit squashed up. I sat in front. I told the driver not to drive that fast because I was scared. Every time I moved, I felt those cramps.”* (Patient 5)

“*That day I walked from work to the train station and then I got a taxi there that dropped me close to home. I told myself, I must just try not to collapse. It was far and very hot.”*

(Patient 6)

#### Inadequate supply of pre-hospital emergency services

An overarching theme reported by all the participants stated that the EMS are not always a feasible option for reaching a hospital due to the often-sub-acute presentation of the condition, or the limited EMS resources within the public sector.

*“There are delays with EMS especially after hours, at weekends, and at month's end when there is an influx of trauma cases.”* (Surgeon 5)

*“There are ambulances, but they only have one in the area. It is better to get to the hospital with your own transport because you are going to wait for a long time."* (Patient 4)

Another dominant theme reported by the patients and surgeons was that EMS often have difficulty locating patients from informal settlements without fixed addresses. Violence against EMS staff is also becoming increasingly prevalent; therefore, they will not enter dangerous areas without a police presence accompanying them.

“*The EMS was previously robbed in our area and were hurt by the people who requested the callout.”* (Patient 5)

### Barriers related to healthcare facility delays

#### Proximity of healthcare facilities

The geographical positioning of the healthcare facility can impede access to care. Logistics of where the nearest EMS is relative to the hospital requiring an inter-facility transfer can lead to delays in the care pathway.

“*The patient might be in [A], and the doctors there would call the EMS who is at [C]. The EMS then needs to go back to [A] to bring that patient to [B]. It is not uncommon to wait for the patient for four to six hours after the referral was made.”* (Surgeon 6)

#### Surgical knowledge and capacity at lower-level facilities

Surgical inefficiencies within the health system and inter-facility transfers can influence the timely administration of healthcare. This includes the efficiency of the referral process.

“*Often doctors do not necessarily know what services the next level can offer. This leads to incorrect referrals and long delays as patients must sit in the queue again, get triaged, and then be referred.”* (Surgeon 1)

Surgeons felt that there was a need to reduce service pressures and increase efficiencies at higher-level hospitals. Referral guidelines are also required for when an individual presents with appendicitis-like symptoms.

*“What we have tried to do is surgical outreach to enable minor procedures to be done in the periphery so that our turnover can be quicker.”* (Surgeon 4)

*“I think, maybe some guidelines for the lower-level facilities to ensure earlier referrals would be useful. Also in the emergency department, red flagging patients who have abdominal pain and sepsis and referring them to us.”* (Surgeon 2)

A dominant theme reported by the surgeons was limited hospital resources in the public health system as an important barrier to receiving appendectomy. This includes a lack or shortage of sufficient and adequately trained staff, a shortage of EMS, no laboratory on-site, and bedspace issues. Another hospital resource that can be a barrier is theatre accessibility which can be limited due to cases of a higher priority taking preference. Appendectomy is often reprioritised when different specialities compete for theatre time within the same facility.

*“Many of the interventions or treatments that can be done at the base hospitals should be done by them. This includes administering early intravenous antibiotics, or imaging. If it is a clear-cut appendicitis that needs surgery, it needs to* be referred to us. *Bedspace is a limiting factor because majority of the time we are running at 90 to 100 % capacity."* (Surgeon 4)

#### Healthcare facility's ability to diagnose and manage appendicitis

A dominant theme expressed by several surgeons was the inability of healthcare providers to diagnose appendicitis timeously and limited hospital resources can affect when the appendectomy is performed. Several surgeons expressed that certain groups of patients such as females, the elderly, and children can be more challenging to diagnose which can lead to a longer care pathway. In addition, males tend to present with a definitive diagnosis of appendicitis, yet they may still undergo a full clinical evaluation contrary to the surgeon's wishes.

“*Some facilities have limited ability to do blood tests, radiology, etc. We often find that the patient might linger at the lower-level facility while they struggle to make a diagnosis. There are often junior doctors working there too. Based on our data, about 60 % of our appendectomies are complicated cases that were already perforated.”* (Surgeon 6)

*“You may end up with gynaecology, going home, or in a medical ward. Especially with our population, HIV confounds a lot of things. So, the differential diagnosis of appendicitis is just wide and you can end up in another speciality. Certain age groups make other diagnoses more likely.”* (Surgeon 2)

## Discussion

Our study showed that barriers to accessing appendectomy are prevalent in the Western Cape, South Africa. The common barriers were categorised into two themes 1) barriers related to late healthcare facility presentation (key barriers were perceptions of appendicitis-like symptoms, the role of tradition, culture, and beliefs on healthcare-seeking behaviour, personal positions and values, transport inaccessibility and affordability, and lack of ambulance services). Similarly, the second broad theme was barriers related to healthcare facility delays (key barriers were proximity to healthcare facilities, surgical capacity, and the diagnostic and management capabilities of the facility). Evidently, following late presentation, patients may still be at risk of delayed appendectomy after entering the health system, and thus longer length of hospital stays, significant cost implications, and possibly even death. Overall these findings resonate with those identified in similar studies conducted in other LMICs, including several African countries [18].

In our study, an overarching theme reported by the patients and surgeons was that appendectomy patients self-medicate, a finding also reported by other studies [11,[27], [28], [29], [30], [31], [32], [33], [34], [35], [36], [37], [38], [39], [40]]. Some patients may also choose to consume traditional medication before seeking healthcare, which is customary in many South African cultures [41,42]. Our study found that appendectomy patients often do not recognise appendicitis-like symptoms [10,11,14,15,27,30,31,33,35,[37], [38], [39], [40],[43], [44], [45], [46], [47], [48], [49], [50], [51], [52], [53], [54], [55], [56], [57], [58], [59], [60], [61], [62]]. In addition, other studies have demonstrated a complication rate of 30 % in appendectomy cases due to a lack of access to health information (29 %), and self-medicating (30 %) [11,12]. Furthermore, barriers to accessing healthcare facilities were related to socioeconomic and structural factors, such as the inability to pay for and access transportation. Lower socioeconomic communities are often forced to choose between providing for their families and prioritising their health needs. These challenges are not unique to the Western Cape Province. Other studies done in Africa and other LMICs, that looked at reasons for late presentation in appendectomy patients also identified far distances to healthcare facilities as a barrier, especially in rural communities [9,10,12,32,35,46,58,59,[63], [64], [65], [66], [67], [68]].

Distances between healthcare facilities can also affect inter-facility transfers of the appendectomy patient. This is worsened by the high trauma burden in the country, which leads to EMS shortages. In addition, surgeons reported that rural communities are often located at least an hour away from the nearest facility and that inter-facility transfers in rural areas can take at least four to six hours, depending on the location of the EMS. According to Kong *et al*., at Edendale Hospital in South Africa [13], more than half of the appendicitis cases observed were perforated after undergoing inter-facility transfers. These factors all contribute to the experience of the patients and can affect their willingness to seek care in the future [69,70].

Other factors associated with delays in receiving appendectomy included lowered clinical acumen and appropriate surgical care and resource availability within the health system at lower-level facilities. This has been reported in other studies where appendectomy patients were initially misdiagnosed due to varying clinical expertise, and an overall lack or shortage of healthcare providers [35,66,[71], [72], [73], [74], [75], [76]]. Many South Africans opt to pay out-of-pocket for private healthcare due to a lack of medical insurance [19]. A critical lack of resources within healthcare facilities [12,14] due to a lack of funding is a major obstacle to effective healthcare service provision in many African countries and other LMICs [10,33,35,66,[77], [78], [79], [80], [81]]. Several countries also have a huge disparity between skilled and experienced healthcare providers employed in the private sector compared to the public sector [[72], [73], [74], [75], [76],^82^].

Our study had some limitations. The conclusions that can be made from our findings are limited by the small sample size. However, given the qualitative paradigm, the findings are not meant to be generalizable to other contexts but aid in shedding light on the barriers that those living and working in low-and middle-income settings face in this regard. Our findings do align with similar studies conducted in other similar settings. It was not possible to determine whether the reported barriers would have directly resulted in delays in accessing appendectomy as our study was qualitative. Patient recall bias may have influenced reported perceptions and beliefs regarding the services rendered when they had an episode of appendicitis.

## Considerations for future policy and practice

There is a need for improved health literacy among communities. Our findings and the literature also suggest that it may be beneficial to incorporate traditional healers and the advice that they give, into the care pathway of the appendectomy patient [83,84]. Health literacy among communities could also be improved through awareness campaigns and the dissemination of health information in the waiting rooms of healthcare facilities. Surgeons from our study suggested surgical outreach as a means to enable lower-level facilities to manage minor procedures, thus lessening the burden at higher-level facilities and the need for inter-facility referrals [85,86]. Ultimately there is a need for increased resource allocation across the health system, especially concerning EMS. We recommend that future studies examine the feasibility and effectiveness of implementing these measures.

## Conclusion

There is a gap in the knowledge of barriers to accessing appendectomy in the different provinces of South Africa, especially in the Western Cape. Our study highlighted that appendectomy patients experience similar barriers to those reported elsewhere in South Africa and other LMICs. Improved health literacy in communities is required to ensure timely healthcare-seeking behaviour for appendicitis and other emergency conditions. Efforts are also needed to ensure access to affordable and available transport options, and all facilities need to be better equipped to diagnose and treat appendicitis. This can be achieved through upskilling and augmenting human and other resources, which will require the support of the government and other relevant stakeholders.

## Dissemination of results

Results from this study will be presented to the scientific and local communities through conference presentations and poster presentations.

## Authors’ contribution

Authors contributed as follows to the conception or design of the work; the acquisition, analysis, or interpretation of data for the work; and drafting the work or revising it critically for important intellectual content: *JL 35 %; RE 30 %; KC 25 % and PN 10 %*. All authors approved the version to be published and agreed to be accountable for all aspects of the work.

## Declaration of competing interest

The authors declare that they have no known competing financial interests or personal relationships that could have appeared to influence the work reported in this paper.

## Appendix Supplementary materials

Image, application 1
