# Supplementary material for: Barriers to accessing appendectomy in the public sector health system in the Western Cape Province, South Africa
Source: Afr J Emerg Med. 2024 Nov 9;14(4):499–505. doi: 10.1016/j.afjem.2024.10.221 (PMC11585650; doi:10.1016/j.afjem.2024.10.221)
Supplement: Supplementary file 1 [file mmc1.docx]

Appendix 1:

Table 1. Participants demographics including age, gender, and race

| Participant | Age | Gender | Race |
| --- | --- | --- | --- |
| Patient 1 | 46 | male | coloured |
| Patient 2 | 71 | female | coloured |
| Patient 3 | 47 | male | black |
| Patient 4 | 45 | male | white |
| Patient 5 | 36 | male | coloured |
| Patient 6 | 26 | male | coloured |
| Patient 7 | 45 | female | coloured |
| Patient 8 | 33 | male | coloured |
| Patient 9 | 36 | male | coloured |
| Patient 10 | 21 | male | coloured |
| Patient 11 | 38 | male | coloured |
| Patient 12 | 22 | male | coloured |
| Surgeon 1 | 34 | female | white |
| Surgeon 2 | 46 | female | coloured |
| Surgeon 3 | 42 | male | white |
| Surgeon 4 | 37 | male | white |
| Surgeon 5 | 43 | male | white |
| Surgeon 6 | 57 | male | white |
